# COVID-19: Implications for Advanced Care Planning and End-of-life Care

**DOI:** 10.5811/westjem.2020.6.48049

**Published:** 2020-07-21

**Authors:** Mishal Reja, Jay Naik, Payal Parikh

**Affiliations:** Rutgers Robert Wood Johnson Medical School, Division of Internal Medicine, Department of Medicine, New Brunswick, New Jersey

Dear Editor:

It was 4 am when the hospital admitting medicine service phone rang. “Ten patients with suspected COVID-19 were sent from a nursing home; it’s possible that they all may need intensive care unit [ICU] beds. How many beds are available right now?” I will never forget the series of events that followed. The urgency was palpable as evidenced by the frenzy of navigating the emergency department, careful donning and doffing of personal protective equipment, and rapid-fire triaging of each patient. It was 6 am when several more patients from that same nursing home arrived. The nasal cannulas turned into non-rebreathers, which quickly transitioned to high-flow nasal cannulas. The next obvious step was intubation. But one question persisted in our minds: “Are we doing the right thing?”

## INTRODUCTION

The rapid global spread of coronavirus disease of 2019 (COVID-19) has resulted in considerable emotional and physical distress in a time of limited medical resources. As healthcare systems have been pushed to the brink, advanced care planning and end-of-life life discussions are of the utmost importance. Palliative care is at a unique vantage point to help treat symptomology and provide guidance. Due to resource limitations, we aim to outline pressing, palliative care needs from a critical care and emergency medicine standpoint.

### Advanced Care Planning and End of Life Discussions

Advanced care planning involves the process of having patients and families make decisions about their last phase of life prior to losing decisional capacity.^1^ Unexpected death is a common event during COVID-19 illness. ICUs around the globe are being filled to and/or past capacity. Studies show that patients ≥ 65 years have a 3.7× greater risk of mortality, and pre-existing cardiovascular and cerebrovascular disease also contribute to increased mortality.^2^ The disease is likely to be fatal for elderly and frail individuals with pre-existing conditions. For these patients, hospitalization and aggressive interventions in critical care units are unlikely to improve quality of life or survival. In a pandemic, the escalation to critical care and aggressive, life-saving measures is rapid with little time for appropriate planning. It would be beneficial to implement early advanced care planning in the outpatient setting for high-risk patients to stay home with hospice care or home health services. Prior studies have shown that patients with outpatient palliative care consultations were 2.5 times more likely to enroll in hospice, and they had lower rates of aggressive medical interventions.^3^

### Grief Considerations

The COVID-19 pandemic has disrupted the grief process for families and friends who have experienced the passing of a loved one from COVID-19. Family visits are usually limited or prohibited, and funerals and burials are held remotely. Complicated grief, secondary traumatic stress, and moral distress is to be expected.^4^ We must also bear in mind that families may have had multiple losses and may be in social isolation from self-quarantine. Maladaptive psychological processing will likely exacerbate post-loss bereavement, exacerbating depression, anxiety, anger, blame, and helplessness. It will be especially important to connect families to resources and self-care practices that they will need.

### Emerging Technology and Artificial Intelligence

Family members of critically ill COVID-19 patients with a poor prognosis face challenging scenarios. Anecdotally, those who have been resistant to withdrawing aggressive medical care demonstrate a lack of understanding of the disease process combined with severe psychological distress, which is exacerbated by their inability to be at the bedside. Several modalities may help engage family members in a dialogue for advanced care planning. These conversations should take place in an outpatient setting by primary care physicians prior to the need for hospitalization for high-risk patients. Evidence-based communication educational curricula can be implemented to coach providers to have difficult conversations if palliative care is unavailable. Artificial intelligence and telehealth technology can assist palliative and primary care providers to monitor and treat end-of-life symptoms at home. Furthermore, mobile health apps have been shown to be successful in goals-of-care discussions for oncology patients,^5^ and these can be adopted for high- risk patients at risk for COVID-19, such as the elderly, those with multiple comorbidities, or those residing in nursing homes. Video messaging with patients and families is often used, and further research needs to be done in this area.

## CONCLUSION

End-of-life discussions are a daunting task. However, effective and empathetic goals-of-care discussions before a crisis situation are particularly important.^6^ Telehealth is a valuable tool to facilitate these discussions, and further research in this area is needed.^7^ COVID-19 has resulted in high mortality and morbidity rates in at-risk populations, and it is imperative to facilitate these discussions early on during this pandemic.
